# The core of the matter: The catalytic core of a cellulose synthase contributes to endomembrane trafficking and plasma membrane dynamics

**DOI:** 10.1093/plcell/koad114

**Published:** 2023-04-29

**Authors:** Carlisle Bascom

**Affiliations:** Assistant Features Editor, The Plant Cell, American Society of Plant Biologists, USA; Department of Cell and Developmental Biology, University of California San Diego, La Jolla, CA 92093, USA

In plants, cell wall biosynthesis is synonymous with cell growth. Plant cell walls are composed of interacting networks of polysaccharides, including pectins, hemicelluloses, and cellulose. Cellulose is the strongest component of primary cell walls and is made of β-1,4-linked glucose chains. This glucose is polymerized at the plasma membrane by CELLULOSE SYNTHASE (CESA) protein complexes. At the catalytic core of each CESA protein is a glycosyltransferase type II (GT2) domain that synthesizes uridine diphosphate glucose into β-1,4-glucans (Purushotham et al. 2020). There are several conserved amino acid motifs within the catalytic core, including DDG, DXD, TED, QXXRW, and interfacial helix 3 (IF3) motifs (Turner and Kumar 2018). Previous work in *Arabidopsis thaliana* (Arabidopsis) identified amino acid substitutions in or around these motifs that resulted in CESA proteins resistant to Endosidin 20, an inhibitor of cellulose synthesis (Huang et al. 2020) (Fig.). Interestingly, Endosidin 20 treatment inhibits both CESA motility at the plasma membrane and CESA trafficking to the plasma membrane (Huang et al. 2020). These results suggest that the above-conserved motifs in CESAs function in endomembrane trafficking as well as CESA catalysis. In this issue, **Lei Huang and colleagues (Huang et al. 2023)** report on amino acid substitutions in conserved motifs of AtCESA6 that shed light on their role in the function of AtCESA6 in facilitating plant cell growth (Figure).

**Figure.**
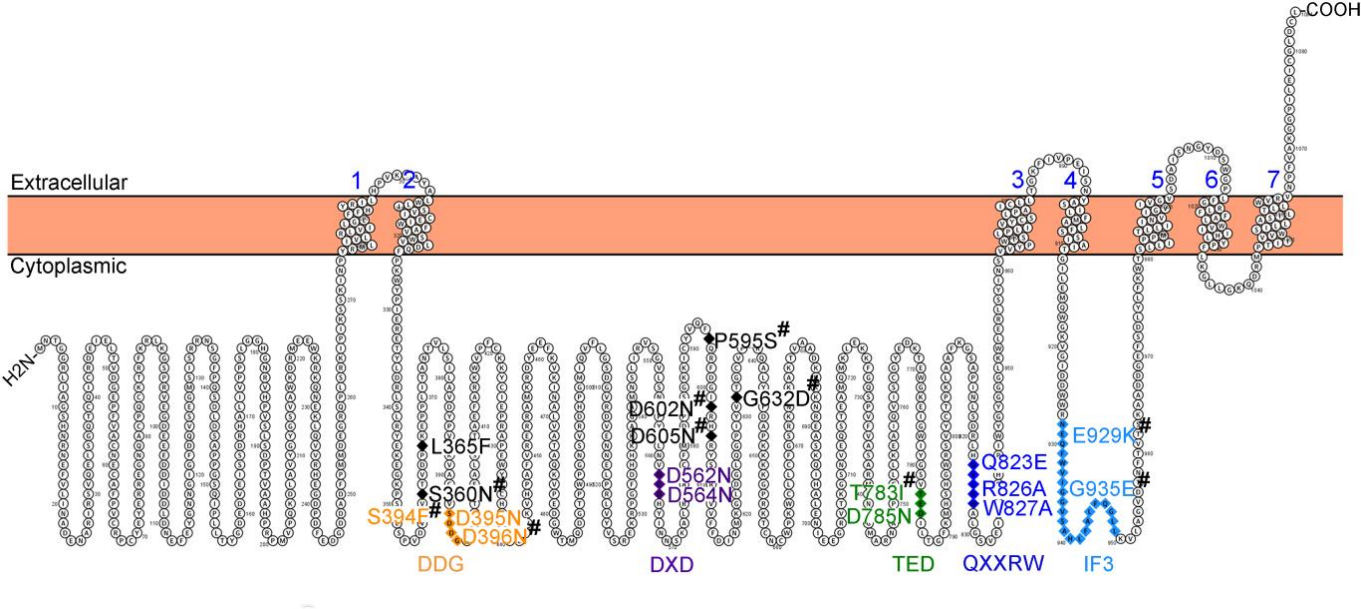
Schematic of mutations tested in this study mapped onto the topology of AtCESA6. Colored text highlights conserved motifs, black text indicates neighboring mutations mutants. #, mutations identified in an Endosidin 20 resistance mutant screen (Huang et al. 2020). Adapted from Huang et al. (2023), Fig. 1.

To test how motifs in the core of AtCESA6 contribute to protein function, the authors complemented *cesa6* mutant plants with mutagenized *YFP-AtCESA6* expressed via the native promoter. They selected 18 single amino acid substitutions in or near conserved motifs within the GT2 domain. Ten of these mutations were identified in a screen for Endosidin 20–resistant plants (Huang et al. 2020). The additional 8 targeted the DDG, DXD, TED, or QXXRW motifs (Figure). The *cesa6* mutation results in seedlings with short dark-grown hypocotyls and reduced cellulose content (Fagard et al. 2000). Therefore, the authors tested these parameters in mutagenized CESA6 complementation lines. Additionally, the authors measured YFP-CESA6 particle movements across the plasma membrane, YFP-CESA6 accumulation in the Golgi apparatus, and protein levels in subcortical small CESA-containing compartments. Further, they conducted fluorescence recovery after photobleaching assays at the plasma membrane and the Golgi. These experiments allowed the authors to evaluate the effect of each mutation on CESA6 function, motility, and endomembrane trafficking.

The authors generated substantial data for each CESA mutant and employed principal component analysis to identify relevant trends in the data. In this way, they were able to categorize the 18 mutations into 2 groups. Group I includes mutations in or near the DDG motif as well as those in the IF3 motif. Mutations in this group cause trafficking defects because mutant proteins were largely retained in the Golgi and there was less YFP-CESA6 signal at the plasma membrane. Group II includes mutations in the DXD, TED, and QXXRW motifs. Mutations in these motifs likely lead to protein misfolding or inhibition of oligomerization of CESA complexes in the endoplasmic reticulum or Golgi.

Taken together, the work presented by Huang and colleagues provides compelling insights into how the catalytic cores of CESAs contribute to CESA trafficking and function. This work provides a launchpad from which we can ask transformative questions: Are there amino acid substitutions that enhance CESA trafficking and function? Do similar mutations in other members of the CESA gene family have a similar effect? And are the effects of the mutations investigated here the same across the plant lineage?
